# Proposed Nutrient Standards for Plant-Based Beverages Intended as Milk Alternatives

**DOI:** 10.3389/fnut.2021.761442

**Published:** 2021-10-20

**Authors:** Adam Drewnowski, Christiani Jeyakumar Henry, Johanna T. Dwyer

**Affiliations:** ^1^Center for Public Health Nutrition, University of Washington, Seattle, WA, United States; ^2^Clinical Nutrition Research Centre, Singapore Institute of Food and Biotechnology Innovation, Singapore, Singapore; ^3^Frances Stern Nutrition Center Tufts Medical Center and Jean Mayer USDA Human Nutrition Research Center on Aging, Department of Medicine, School of Medicine and Friedman School of Nutrition Science and Policy at Tufts University, Boston, MA, United States

**Keywords:** plant-based beverages, milk alternatives, nutrient standards, added sugar, fortification, Codex Alimentarius, Food and Drug Administration, standards of identity

## Abstract

**Background:** Plant-based beverages (PBB) that are marketed as alternatives to cow milk are gaining in popularity worldwide. Nutrient quality of PBB can be highly variable.

**Objective:** To develop a set of voluntary or mandatory nutrient standards for the PBB product category in order to assist innovation and guide product development and reformulation.

**Methods:** The present goal was to develop standards for PBB energy content, minimum protein content and quality, maximum content for added fat, sugar, and salt, and to suggest fortification levels for selected vitamins and minerals. The standards were based on dietary recommendations and guidelines and current practices of federal agencies in the US.

**Results:** The proposed energy and nutrient content for PBB milk alternatives are maximum 85–100 kcal energy per 100 g; a minimum for 2.2/100 g of high-quality protein, low content of saturated fat (<0.75/100 g) and added sugar (5.3–6.25/100 g) and consistent fortification with calcium, vitamins A, D, B-2, and B-12 at levels comparable to those found in cow milk (1%). Ideally, the protein content ought to be increased (2.8/100 g) and added sugar content reduced even further (2.7–3.1/100 g) for “best of class” products. These proposed standards were applied to the 641 existing PBB products listed in the 2018 version of the USDA Branded Food Products Database (BFPDB). The standards were met by <5% of the PBB on the US market.

**Conclusion:** Often viewed as equivalent to milk in nutritional value, many PBB are often low in protein and are fortified with varying amounts of calcium, and vitamins A and D. Nutrient standards for this category should be adopted by the food industry, by public health regulatory authorities, and by standardization bodies such as the Codex Alimentarius.

## Introduction

Plant-based beverages (PBB) that serve as milk alternatives are a rapidly growing market segment (1, 2). As shown in past studies (3–5), both energy content and nutritional quality of different-source PBB can be highly variable. Nevertheless, many consumers believe that PBB milk alternatives offer the same nutritional value as dairy milk, containing the same nutrients but no lactose and less saturated fat (6–8).

Ensuring that the new PBB products are not nutritionally inferior to milk but provide adequate nutritional value becomes a matter of public health concern. Developing a set of proposed nutrient standards for the emerging PBB category would be of value to regulatory agencies, standardization bodies, and to the food industry in the US, the European Union, and elsewhere. Such standards, applied to existing and to new PBB products, would inform best manufacturing practices and/or any potential PBB nutrient content claims. Thus, far, the only nutritional requirements for the PBB product category are linked to voluntary nutrient profiles and front-of-pack labels. The European Nutri-Score, Australia-New Zealand Health Star Rating, and Choices International have listed criteria for beverages, including PBB to achieve a good score (3).

Proposed guidance on the labeling of plant-based milk alternatives is currently under development at the Food and Drug Administration in the US. The Guidance for Industry document is expected to appear in draft or final form by the end of June 2022 (9). The FDA has raised previous concerns about the potential for confusion between PBB and cow milk. At this time, PBB products formulated from nuts, legumes, grains, and seeds are allowed to be called plant “milks” in the US. With some exceptions, notably for almond and coconut milks, the use of dairy terms to describe non-dairy foods and beverages is not permitted in the European Union (10).

The present position is that those PBB products that are specifically marketed as milk replacements ought to be broadly equivalent to cow milk in terms of nutritional value, consistent with the US Code of Federal Regulations (11, 12). In the US, milk is the principal source of dietary calcium and vitamin D and contains multiple minerals critical to bone health (13). Milk is also an important source of dietary potassium, iodine, riboflavin, and vitamin A (13). The USDA recommends 2.5 cups of dairy for 4–8 y and 3 cups for older children daily, noting that an 8 oz glass of milk provides 40% of daily protein needs for a child 4–8 years of age, 50–60% of daily calcium needs, and 10% of daily potassium needs. Accounting for only 14.2% of dietary energy, milk, cheese, and yogurt contributed 23.7% of protein, 61.6% of calcium, 65.8% of vitamin D, 38.5% of vitamin A, and 38.3% of vitamin B12 to US children's diets (14).

Unsweetened low-fat (1%) milk provides 3.3/100 g of protein; about 5/100 g of lactose, the naturally occurring sugar, and 0.95/100 g of fat, of which 0.57 g is saturated fat (15). By contrast, PBB milk alternatives generally contain about 1/100 g plant protein and can vary widely in their content of added sugar and saturated fat (3, 4). These products are typically fortified with varying amounts of calcium, vitamin D, and vitamin A and less often with vitamin E, vitamin B-12, and zinc (4). At this time, pediatricians do not recommend PBB milk replacements for children (16) largely because of inadequate protein content, low protein quality, and other nutritional shortcomings (16–18). No standards of identity for PBB are available in the US at this time.

## Proposed PBB Nutrient Standards

Any proposed nutrition standards for PBB milk alternatives would need to address, at a minimum, desired energy content, optimal amounts and quality of the plant protein(s), limits on total or saturated fat, added sugar and salt, and the overall strategy for fortification with vitamins and minerals.

Since many PBB are positioned as milk alternatives, unsweetened low fat (1%) milk was the first obvious point of comparison. The proposed standards were expressed per 100 g, to better align with standard methods of listing nutrient composition of foods. Although the FDA food labels in the US are based on serving sizes, known as Reference Amounts Customarily Consumed (9), suggested values per 100 g are closer to food labeling standards used in the European Union. The proposed standards for PBB milk alternatives are summarized in Table 1.

**Table 1:** Proposed energy content and minimum nutrient criteria for PBB milk alternatives.

**Nutrient**	**Milk 1% fat per 100 g**	**Proposed nutrient standards** **per 100 g PBB**	
		**Children (4–12 y)**	**Adults (>12 y)**
Energy (kcal)	43	<85	<100
Protein (g)	3.38	>2.2	>2.2
Best of class protein (g)	–	>2.8	>2.8
Protein quality (PDCAAS)	1.0	>0.9	>0.8
Total/added/free sugar (g)	4.96	<5.3	<6.25
Best of class sugar (g)	–	<2.7	<3.1
Saturated Fat (g)	0.57	<0.75	<0.75
Sodium (mg)	39	<120	<120
Calcium (mg)	126	>15% DV/200 g serving	>15% DV/200 g serving
Riboflavin (B-2), (mg) Vitamin D (IU) Vitamin B-12 (mcg) Vitamin A, rae (mcg)	0.14 42.4 0.6 58	>15% DV/200 g serving	>15% DV/200 g serving
Fiber (g)	0	Optional	Optional
Carbohydrates (g)	5.18	Optional	Optional
Potassium (mg)	159	Optional	Optional

*DV, daily value; PDCAAS, protein digestibility corrected amino acid score*.

### Energy Per 100 g

The desired maximum energy content for PBB milk replacements was set at 85–100/100 g or 170–200 kcal per serving. These energy levels correspond roughly to 10% of daily energy intakes estimated at 1,700 kcal/d for children and 2,000 kcal/d for adults. Many initiatives to limit the marketing and advertising of snacks to children have also set the limit at 170–200 kcal per serving. The USDA Smart Snacks in Schools regulation that applies to foods sold on school campuses during the day sets the limits for vending machine snacks at ≤200 calories (19). The recent limits set by Google for snacks and treats in the United Kingdom (UK) and the European Union (EU) use <150 kcal per serving (20).

In the US, most people have 2–3 snacks per day so that snacks contribute a total of 23% of energy to the diet (21). Most snacks are consumed in the afternoon/evening, or at night. Between-meal snacking is less common in France, with snacks contributing only 12% of dietary energy (22). Most milk is consumed at breakfast in both France and the US. By way of comparison, a typical breakfast contributes about 18% of daily energy in the US and ~18–21% energy (the equivalent of two between-meal snacks) in France, Ireland, Spain, Canada, and Australia- New Zealand (23). There are limited data on the contribution of breakfast to the daily energy intakes in parts of Asia, Africa, Latin America, and the Middle East.

### Protein: Amount and Quality

Milk is one of the main contributors to daily protein intakes for children (14, 15). The protein content of 1% dairy milk is about 3.3 g/100 g. By contrast, the protein content of most PBB milk alternatives is generally below 1 g/100 g (4). Expanding beyond those from soy, almond, and coconut PBB products now include those sourced from pea, oats, quinoa, and rice. Only pea and soy milks in the US have a protein content that approximates that of cow milk (4).

The proposed minimum protein content for PBB milk replacements was set at 2.2 g of protein per 100 g. However, our aspirational target for “best of class” products was 2.8 g/100 g or 5.6 g protein per 200 g serving, corresponding to 11.2% of daily value (DV). The higher goal was strongly recommended for those PBB that might be marketed as milk alternatives or replacements in low- and middle-income countries.

Concern with protein quality from plant sources was another issue. In the US, the FDA requires adjustment for protein quality for products that are marketed to children under 4 y of age and those that make a protein claim (24). The Protein Digestibility Corrected Amino Acid Score (PDCAAS) is the standard measure of protein quality (25). While protein requirements differ for children and adults, PDCAAS close to 1.0 can satisfy growth requirements for children. Milk proteins have a PDCAAS score of 1.0 (25, 26).

With the exception of soy proteins (PDCAAS 0.91–0.95), individual plant proteins have lower PDCAAS scores (<0.9). Our position was that a PDCAAS score of 0.8–0.9 could be achieved by blending cereal- and legume-based proteins (1, 25, 26). A judicious combination of two or more plant-based proteins would complement the essential amino acids notably lysine, isoleucine and methionine (27). For example, many plant-based meat alternatives use soy protein but others include blends of wheat, pea, rice and bean proteins for a total plant protein content of ~16/100 g. Given advances in food technology, blending plant proteins from diverse food sources is feasible and it should lead to a PDCAAS between 0.95 and 1.0.

In the US, protein claims require >10% DV (i.e., 5 g) of protein per serving for a “good source” claim and >20% DV (i.e., 10 g) of protein per serving for an “excellent source” claim. In the EU, 12 and 20% of energy from protein are required for the “source of” and “high in” claims, respectively.

The PDCAAS has been widely accepted since its adoption in 1991 by the Food and Agriculture Organization of the United Nations (FAO) and the World Health Organization (WHO) (26). However, the FAO now favors the DIAAS (Digestible Indispensable Amino Acid Score), which is reported to better account for the bioavailability of amino acids but it is not yet fully operational, since no data for food products are as yet available (28). The US Food and Drug Administration continues to use PDCAAS for regulatory purposes.

### Added Sugar Content

The proposed goals for added sugars are consistent with the US and international guidelines. In the US, the 2020 Dietary Guidelines for Americans recommended reducing added sugars to no more than 6% daily energy. Similarly, the World Health Organization (WHO) recommendation has been for 10% of energy from added sugars, with 5% proposed for the near future. The Scientific Advisory Committee on Nutrition in the UK currently recommends only 5% of energy from added sugar (29).

**Figure 1:**
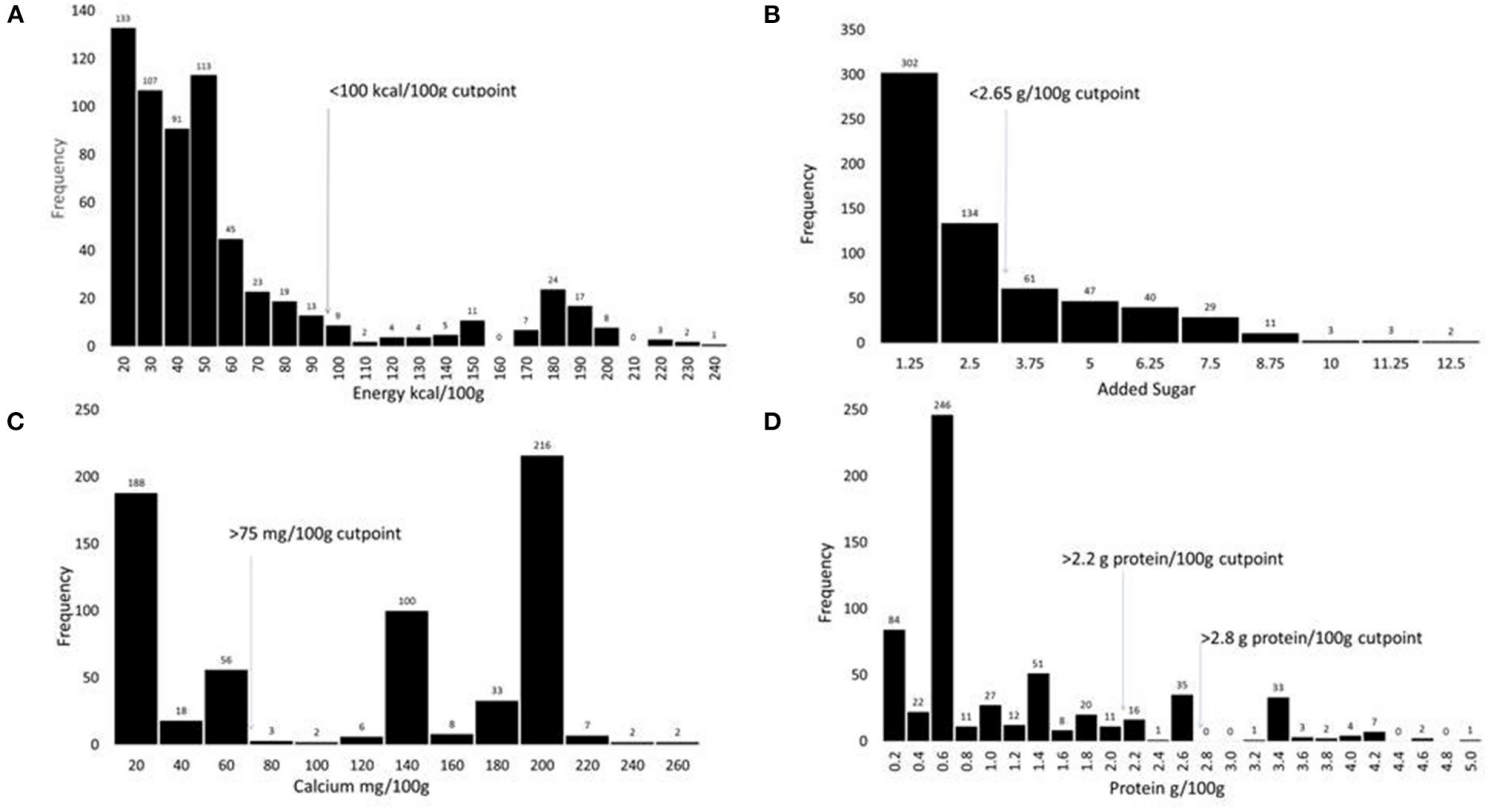
Histograms showing the distribution of PBB products (*n* = 641) according to the content of energy in kcal/100 g **(A)**, added sugar in g/100 g **(B)**, calcium in mg/100 g **(C)**, and protein in g/100 g **(D)**.

The proposed amount of added sugar in PBB should approximately match the level of naturally occurring sugar in milk, that is ~5 g/100 g. Milk contains no soluble or insoluble fiber. The proposed maximum amounts for added sugar were therefore 5.3 g/100 g for children and 6.2 g/100 g for adults. This would place the PBB slightly above milk but at the lower end of the PBB range (see Figure 1 panel B). The aspirational longer-term goal for “best of class” products was to reduce added sugar even further to 2.7 g/100 g for children and 3.1 g/100 g for adults. Those amounts would be equivalent to about half of the level of lactose in milk.

The type and the amount of added sugar in the PBB may have regulatory implications. In the case of beverages, total sugars, added sugars and “free” sugars are essentially the same. In the US, the back-of-pack Nutrition Facts Panel is now required to include added sugars (29). The term “added sugar” refers to caloric sweeteners (sugars) added in the course of manufacturing and processing and includes cane, beet and corn derived sweeteners. Honey and fruit syrups and concentrated fruit and vegetable juices are treated as added sugars by the FDA (29). The term “free sugars” used by the WHO, refers to all monosaccharides and disaccharides added to foods by the manufacturer, cook or consumer, plus the sugars that are naturally present in honey, syrups and fruit juices. In the UK, sugars naturally present in fruit and vegetables that have been blended, pulped, puréed, extruded or powdered are treated as “free sugars” on the basis that the cellular structure has been broken down. That includes juices and syrups. By contrast, sugars naturally present in other types of processed fruit and vegetables (dried, canned, stewed, pressed) fall outside the definition of free sugars (30).

### Fiber

PBB milk alternatives are water-soluble extracts of legumes, oilseeds, oils, or cereals that are homogenized to produce emulsions with an appearance that is close to that of milk (31, 32). Even though consumers may associate PBB with fiber, the fiber content of PBB is generally low, rarely exceeding 0.5/100 g. Milk contains no fiber. While fiber was not a priority for those PBB that were specifically intended as milk alternatives, the addition of fiber could be an added health benefit for other PBB (see section Discussion). Given low fiber intakes in the US and elsewhere, fiber would provide the new generation of PBB with a nutritional advantage. Using soy PBB as the standard, a proposed nutrient standard of 1 or 2 g fiber per 100 mL could be feasible.

### Total and Saturated Fat

The fat content of dairy milk ranges from 0/100 g (skim milk), 1.0/100 g (1% or low-fat milk), 2.0/100 g (2% or reduced fat milk) and 3.5/100 g (3.5% or whole milk). Milk fat is mostly saturated fat. Attempting to remove all the fat in oilseed-sourced PBB to match skim milk poses technical challenges, since many such products derive their milky white appearance and creamy consistency from extracted plant oils held in suspension by stabilizers and emulsifiers (31, 32).

Our perspective was that PBB milk replacements ought to limit saturated fat to <0.75/100 g, corresponding roughly to low-fat (1%) milk. No specific targets were set for mono- or poly-unsaturated fatty acids, such as linoleic acid (18:2 n-6), alpha-linolenic acid (18:3 n-3), or other omega-3 fatty acids that are not present in large quantities in milk. On the other hand, PBB products marketed to vegan consumers could be fortified with long chain omega-3 fatty acids, eicosapentaenoic acid (EPA), and docosahexaenoic acid (DHA).

### Sodium

Cow's milk contains little sodium (<50 mg/100 g on the average). The present recommendation for the maximum amount of sodium in PBB milk alternatives was set at <120 mg/100 g, equal to 6% daily value (%DV). Most PBB products contain very little sodium. However, some plant based concentrated used in the manufacture of PBB (e.g., pea concentrate) may contain small amounts of sodium.

## Strategy for Fortification With Vitamins and Minerals

The present goal was to ensure that PBB milk alternatives were not nutritionally inferior but contained adequate amounts of those vitamins and minerals that were characteristic of the Dairy Group. Reference Daily Intakes (RDIs) for nutrients were used to calculate % Daily Values (%DV) that US consumers see on the Nutrition Facts Panel (11). The Codex Alimentarius uses the term Nutrient Reference Value (NRV) instead of DV.

The proposed minimum standard for fortification with vitamins and minerals was set at 15% Daily Value (%DV) per PBB serving. The adequacy standard in the EU is set at 15%DV, whereas the FDA requires 10%DV per serving to identify a “good source” and 20%DV to identify and “excellent source” of a particular nutrient. However, it must be noted that dairy milk provides much more that 15% DV per serving for calcium, riboflavin, and vitamin D (33). In line with the FDA principle of “substantially equivalent value” (9), fortification targets for those nutrients ought to be raised to levels comparable to those for milk, that is to say above 15%.

The proposed minimum fortification standards are shown in Table 2. PBB milk alternatives ought to be fortified with calcium, vitamin D, vitamin B-2 (riboflavin), vitamin B-12, and vitamin A. Fortification with vitamin E was not viewed as a priority, since milk contains very little vitamin E. Replacing milk with PBB would not impact vitamin E intakes.

**Table 2:** Proposed criteria for vitamin and mineral fortification of PBB per 100 g.

**Compound**	**Fortify**	**Daily value DV or NRV**	**15% DV per 100 g**	**Amount in 100 g of milk, 1% fat**
Calcium mg	Yes	1,000	75	126
Riboflavin (B-2) mg	Yes	1.2	0.09	0.14
Vitamin B-12 mcg	Yes	2.4	0.18	0.61
Vitamin A mcg	Yes	800	60 RAE	58 RAE
Vitamin D2+3 mcg	Yes	15 (IU 600)	1.12 (IU 45)	1.06
Vitamin E mg	No	15	1.12	0.02
Potassium mg	Optional	3,510	258	159
Magnesium mg	Optional	310	23.2	12

*DV, daily value; NRV, nutrient reference value (Codex Alimetarius term); IU, international Units; RAE, Retinol Activity Equivalents*.

In the US, industry standards for fortification range from a minimum of 15% Daily Value (DV) to a maximum 20% of tolerable upper intake (9).

Manufacturers in the US and globally have been fortifying PBB with nutrients closely associated with milk, though sometimes in varying amounts (3). For example, soy milks in the US are characterized by relatively high protein content (3/100 g), high PDCAAS value (>0.90), and consistent fortification with calcium, vitamin A, and vitamin D at levels comparable to milk. Fortified soy milks are therefore assigned by the US Department of Agriculture to the Dairy Group whereas other PBB are not. Dairy milk in the US is fortified with vitamin D; this may not be the case in other countries.

The FDA has approved the addition of vitamin D to PBB that are intended as milk alternatives and made from soy, almond, and coconut, and to edible plant-based yogurt alternatives. Vitamin D was authorized years before for use in soy beverages. Manufacturers can voluntarily add up to 84 IU/100 g of vitamin D3 to milk, 84 IU/100 g of vitamin D2 to PBB milk alternatives, and 89 IU/100 g of vitamin D2 to plant-based yogurt alternatives.

Since milk and dairy products contain very little iron, zinc, magnesium, or thiamin, these were not among the proposed standards. By contrast, plant-based meat analogs are normally fortified with iron, zinc, and vitamin B-12, since those nutrients are commonly associated with meat. For populations at risk for nutrient inadequacy, additional fortification with zinc, magnesium, or thiamin (vitamin B-1) should be considered.

## Testing the Proposed Standards Using USDA Branded Food Products Database

The USDA Branded Food Products Database (BFPDB) is publicly available and can be downloaded from US Agricultural Data Commons (33). The 2018 version of the BFPDB lists 239,089 foods and provides product long name, manufacturer name, energy content, and values for those nutrients (per 100 g) that were listed on the Nutrition Facts Panel. The BFPDB was searched for those PBB that specifically used “milk” in the product name (3, 4). The searches covered alternative spellings (almondmilk) and milk blends. Flavored plant milks with coffee, fruit, and other flavors and cultured milks were included. Products where milk was not the principal name (e.g., milked almonds) were excluded. For PBB blends, electronic ingredient lists were searched to identify the principal plant component. Excluded were PBB with energy density that was below 10 kcal/100 g or above 250 kcal/100 g. The resulting PBB (*n* = 641) were coded as almond (*n* = 273), coconut (*n* = 192), soy (*n* = 101), cashew (*n* = 30), tree nut (*n* = 10), flax/hemp (*n* = 16), pea (*n* = 13), and quinoa and rice (*n* = 6).

## Application of Proposed Standards

Some of the proposed criteria were easier to satisfy than others, based on BFPDB analyses. Table 3 shows that most PBB had energy density below 100 kcal/100 g (553/641) and 535 had energy density below 85 kcal/100 g. The main exceptions were coconut PBB with higher energy density and high saturated fat content.

**Table 3:** Proposed energy and minimum nutrient criteria for PBB milk replacements applied to the PBB in the USDA Branded Food Products Database (*n* = 641).

**Nutrient**	**Nutrient standard per 100 g**	**Number passing (%)**
Sodium (mg)	<120	640 (99.8)
Added/free sugar (g)	<6.25 <5.3	584 (91.1) 550 (85.8)
Energy (kcal/100 g)	<100 <85	553 (86.3) 535 (83.5)
Added/free sugar best of class (g)	<2.65 <2.12	473 (73.8) 441(68.8)
Saturated Fat (g)	<0.75	432 (67.4)
Calcium mg (>15% DV/200 g)	>75	376 (58.7)
Vitamin A IU (>15% DV/200 g)	>200	368 (57.4)
Vitamin B-12 (mcg)	>0.18	219 (34.2)
Protein (g)	>2.2	134 (20.9)
Vitamin D (IU)	>45	113 (17.6)
Protein best of class (g)	>2.8	97 (15.1)
Vitamin B-2 mg (>15% DV/200 g)	>0.09	0 (0)

*The criteria are ranked in order of compliance*.

All PBB (640/641) were below the proposed maximum 120 mg/100 g standard for sodium. A total of 584 PBB met the 6.25/100 g target for added sugar for adults and 550 met the <5.3/100 g target for children. Fewer PBB met the stricter target of 3.12/100 g of added sugar (473/641) for adults and 2.65/100 g for added sugar for children (441/641). Most likely to be sweetened were almond and soy milks; in contrast, coconut milks were unsweetened but high in saturated fat. Soy, pea, and rice PBB milks had the highest added sugar content while coconut, oat and tree nut milks were more likely to be unsweetened (4).

Most PBB contained little or no saturated fat and 432 (67%) met the 0.75/100 g thresholds. Again, coconut milks were the exception and virtually all (191/192 or 99%) had more saturated fat than the proposed standards, and so did 33% of cashew PBB.

For calcium, 376 PBB (59%) contained more than 15% DV per 200 g serving. Fully 368 PBB (57%) were fortified with vitamin A; this included 90% of soy milks and most (78%) of the almond milks. Vitamin B-12 was found in 219 (34%) PBB in amounts exceeding 15%DV. By contrast. Only 102 (16%) PBB contained riboflavin and none in the proposed amounts.

Protein and vitamin D are among the main assets of the Dairy Group. The protein content of PBB was problematic, even before the application of PDCAAS. Only 134 (21%) PBB contained more than 2.2/100 g of protein. Higher level of protein (>2.8/100 g) was found in only 97 (15%) of PBB. Those were all pea milks and 66% of soy milks. Only 113 (18%) PBB were fortified with vitamin D (60% of soy milks). In summary, PBB strong points were low energy density and low added sugar and sodium; however, only few PBB met the proposed standards for protein and vitamin D.

The distribution histograms in Figure 1 show that most existing PBB satisfied the proposed criteria for sodium, energy, and added sugar. Fewer satisfied the proposed fortification standards. Critically, very few existing PBB products met the proposed standards for protein.

Finally, Table 4 shows the effects of progressive application of proposed standards to PBB in the BFPDB. For example, as documented above, most of the PBB readily satisfied the criteria of low energy density and low added sugar. All coconut milks were eliminated by proposed standard for low saturated fat content. The need to meet the proposed standards for calcium eliminated about half of the PBB, reducing the number to 333.

**Table 4:** The application of multiple nutrient standards to the BFPD database of 641 PBB.

**Number of PBB products that pass multiple nutrient standards**								
**PBB type**	**#**	**Energy**, **Sugar**	**Energy**, **Sugar** **Ca**	**Energy**, **Sugar** **Ca** **Protein** **(>2.2 g)**	**Energy**, **Sugar** **Ca** **Protein** **(>2.8 g)**	**Energy**, **Sugar** **Ca** **Protein** **(>2.8 g)** **Vitamin D**	**Energy**, **Sugar** **Ca** **Protein** **(>2.8 g)** **Vitamin D** **Vitamin A**	**Energy**, **Sugar** **Ca** **Protein** **(>2.8 g)** **Vitamin D** **Vitamin A** **Vitamin B-12**
Almond	273	251	185	9	8	0	0	0
Cashew	30	27	11	0	0	0	0	0
Coconut	192	98	23	0	0	0	0	0
Soy	101	89	81	77	54	31	31	30
Flaxhemp	16	15	11	2	2	0	0	0
Treenuts	10	10	7	0	0	0	0	0
Pea	13	10	10	10	10	7	7	0
Quinoa/rice	6	5	5	0	0	0	0	0
Total	641	505	333	99	74	38	38	30

*Ca, calcium; Standards for energy <100 kcal/100 g; added sugar <6.25/100 g; calcium >75 mg/100 g; sat fat <0.75/100 g; protein either >2.2/100 g or >2.8/100 g; vitamin D >1.12 mcg/100 g, vitamin A >200 IU/100 g, vitamin B- >12 45 IU/100 g*.

The most glaring problems were with protein and vitamin D. Imposing the additional 2.2/100 g protein standard reduced the number of compliant PBB to only 99 products, mostly pea, soy and some almond PBB. The more stringent protein standard of 2.8/100 g (still below that of dairy milk) reduced the number of compliant PBB down to 74 items, mostly pea, soy and some almond PBB.

Progressive application of vitamin D fortification standards further reduced the total PBB to 38 (6%) all of which were pea and soy PBB. Applying the standards for vitamin A and vitamin B-12 reduced the number of PBB that complied with the proposed standards to 30 (5%). All of those were fortified soy milks.

## Discussion

There is a great deal of interest in plant-based foods, including PBB milk alternatives. Their nutritional quality has become a matter of public health concern, especially when it comes to child nutrition (16–18). In the US, milk and dairy products are the main food sources of dietary calcium, potassium and vitamin D (13–15). Milk and dairy products made significant contributions to dietary intakes of protein, riboflavin, vitamin B-12, vitamin K, and vitamin A among infants and children (13–15, 34). However, milk and dairy products can also provide added sugars and saturated fats.

The present goal was to suggest a set of standards for PBB nutrient content for voluntary adoption by regulators and the food industry. The proposed standards may inform the development of labeling requirements by regulatory agencies including the US FDA. Ensuring that the new products are not nutritionally inferior is one matter of public health concern.

The proposed nutrient standards were based on the nutritional profile of unsweetened low fat milk (1%). However, the PBB milk alternatives that were screened for nutritional value were of highly variable energy density and differed widely in their protein and micronutrient content. About half of the products listed in the BFPDB were sweetened and most were fortified with variable amounts of calcium, vitamin A, and vitamin D. With some exceptions (soy and pea), the PBB category did not approach the level of protein found in milk. In summary, the proposed standards were met by only 5% of the 641 PBB screened.

The few PBB that were consistent with the proposed standards were selected fortified soy milks that were relatively high in protein, contained little added sugar and saturated fat and were fortified with vitamins D, A, and B-12. In the US, soy milks are typically fortified with calcium, vitamin A and vitamin D, and less often with vitamins B-2 and B-12. Fortified soy milks are classified by the USDA as belonging to the dairy group and are approved for use in the Special Supplemental Nutrition Program for Women, Infants, and Children (WIC).

The low content of mostly low quality protein (apart from soy, pea, and legume cereal blends) was a problem for many PBBs. Of all the nutrients examined, the protein gap between PBB and cow milk was the most difficult to address. Only pea and soy milks, the latter already recognized as part of the Dairy Group by the USDA, were able so satisfy the proposed protein nutrient standards. In general, protein content of the PBB was about 1 g/100 g as opposed to 3.5 g/100 g for cow's milk. It is therefore ironic that PBB milk alternatives benefit from the “halo” of healthy plant protein, even though their protein content is low. Often, PBB milk alternatives are perceived by consumers as “healthier” than milk because, apart from coconut milk, most contain little or no saturated fat. In recent surveys, consumers also valued low energy content, low saturated fat and the presence of vitamin A and vitamin D in PBB milk alternatives (35, 36).

In high income countries, milk and dairy provide calcium, potassium and vitamin D, but they are not the main source of high quality protein. The consumption of meat, poultry, and fish is sufficiently high. By contrast, in low and middle income countries, dairy products (and eggs) rather than meat are often the chief sources of animal protein. Widespread use of PBB milk alternatives in place of milk would be a cause for nutritional concern.

PBB are clearly being marketed as more sustainable and planet-friendly than dairy milk. However, their production involves some complex food technology and processing, so that PBB milk alternatives are for the most part ultra-processed foods (3). A recent analysis of the same BFPDB database of PBB milk alternatives identified 91% as ultra-processed following the application of published NOVA criteria for ultra-processed foods (3). There is a paradox here: plant forward diets cannot move forward without relying on ultra-processed foods. PBB milk replacements and plant based meat analogs benefit for some complex engineering.

The present analyses may inform the formulation of new PBB for optimal nutrient value and guide some regulatory initiatives. The present conclusion was that, except for fortified soy milks, PBB should not be assigned to the Dairy Group in the US (or in recommendations for dairy in dietary guidelines in various countries), until some voluntary standards are developed and adopted by the food industry. Such nutrient standards would be the first step toward developing standards of identity for the PBB milk alternatives.

Of course, not all PBB are intended as milk alternatives. Non-traditional ingredients for the creation of PBB can include cereals (oat, rice, corn, spelt); legumes (peanut, lupin, cowpea); tree nuts (almond, coconut, hazelnut) seeds (flax, hemp, sunflower); and pseudo cereals (quinoa, teff, amaranth). Thus far, many of the PBB have been based on extracted plant oils and have been positioned and treated as milks because of their opacity and white color. However, evolving food technology may lead to the creation of PBB with distinct nutritional signatures.

Nutrient density of new generation PBB could be improved through the use of multiple plant proteins, healthy fats, new sweeteners, and fortification with vitamins and minerals to address local needs. While no specific standards are proposed, the general principle should be that those PBB have at least one positive nutritional attribute, in addition to limiting energy, total and added sugar, sodium and saturated fat. There are opportunities to create PBB from under exploited cereals and legumes including quinoa, amaranth, fava beans, chickpea, kidney beans, and azuki beans. Novel plant lipids include those from algal, rice bran oil, jojoba, sal seed, and shea butter. New PBB could also contain bioactive compounds, including flavonoids, polyphenols and sterols. However, such products ought not to be called “milk.”

One limitation of the present screening analysis is that it was based primarily on PBB available in the US and not worldwide. The nature of the US food supply is rapidly evolving with new products being introduced daily. Data on PBB nutrient content may be different in the future or across different countries. Accurate and updated nutrient composition databases of branded food products for high income and for lower income countries are essential for the continuing monitoring of the processed food supply. In particular, the electronic ingredient lists, a new addition to the research toolbox (3, 4, 33) were particularly valuable and will continue to be useful for research and for regulatory purposes.

## Conclusion

PBB products that are marketed as milk alternatives would benefit from clear standards of identity. The FDA approach to good manufacturing practices (GMP) is to provide recommendations for the formulation of food products with healthful nutritional credentials. By enforcing standards of identity, the FDA could take another regulatory step. This report proposes some recommendations that could serve as a starting point for such discussions.

## Data Availability Statement

Publicly available datasets were analyzed in this study. This data can be found here: https://data.nal.usda.gov/dataset/usda-branded-food-products-database.

## Author Contributions

AD, CH, and JD: conceptualization, methodology, and writing—review and editing. AD: formal analysis, data curation, writing—original draft preparation, and project administration. All authors have read and approved the published version of the manuscript.

## Funding

The review of nutrient standards for the plant-based beverage product category and analyses of publicly available USDA Branded Food Products Data Base in the US were supported by Nestlé.

## Conflict of Interest

AD is the developer of the Nutrient Rich Food (NRF) index, a nutrient profiling model and has received grants, contracts and honoraria from entities, both public and private, with an interest in nutrient density of foods, including both dairy products and plant based dairy alternative beverages. AD is a member of the Nestlé Scientific Advisory Board. CH and JD have consulted Nestlé in the past. JD is a member of the scientific advisory boards of McCormick Science Institute, the Mushroom Council, and Bay State Milling. She holds stock in several food and drug companies.

## Publisher's Note

All claims expressed in this article are solely those of the authors and do not necessarily represent those of their affiliated organizations, or those of the publisher, the editors and the reviewers. Any product that may be evaluated in this article, or claim that may be made by its manufacturer, is not guaranteed or endorsed by the publisher.
